# Initial risk factors, self-compassion trajectories, and well-being outcomes during the COVID-19 pandemic: A person-centered approach

**DOI:** 10.3389/fpsyg.2022.1016397

**Published:** 2023-02-08

**Authors:** Hali Kil, Eric Lacourse, Geneviève A. Mageau, Mathieu Pelletier-Dumas, Anna Dorfman, Dietlind Stolle, Jean-Marc Lina, Roxane de la Sablonnière

**Affiliations:** ^1^Department of Psychology, Université de Montréal, Montreal, QC, Canada; ^2^Department of Psychology, Simon Fraser University, Burnaby, BC, Canada; ^3^Department of Sociology, Université de Montréal, Montreal, QC, Canada; ^4^Department of Psychology, Bar Ilan University, Ramat Gan, Israel; ^5^Department of Political Science, McGill University, Montreal, QC, Canada; ^6^École de Technologie Supérieure, Université du Québec, Montreal, QC, Canada

**Keywords:** pandemic, risk factors, self-compassion, well-being, latent class analysis, latent class growth analysis

## Abstract

**Introduction:**

We investigated whether initial risk classes and heterogeneous trajectories of self-compassion over the course of the pandemic may impact well-being outcomes 1 year into the pandemic.

**Methods:**

A large, representative sample of Canadians (*N* = 3,613; 50.6% women) was sampled longitudinally over 11 waves (April 2020–April 2021), using a rolling cross-sectional survey design. Analyses were conducted in three steps: (1) latent class analysis to identify heterogeneity in risk factors (sociodemographic, cognitive-personality, health-related) early in the pandemic, (2) latent class growth analysis (LCGA) to identify longitudinal self-compassion trajectories, and (3) GLM to examine effects of risk factor classes and self-compassion trajectories, as well as their interaction, on later well-being (mental health, perceived control, life satisfaction).

**Results and Discussion:**

Four risk factor classes emerged, with 50.9% of participants experiencing low risk, 14.3% experiencing multiple risks, 20.8% experiencing Cognitive-Personality and Health risks, and 14.0% experiencing sociodemographic and Cognitive-Personality risks. Four self-compassion trajectories also emerged, with 47.7% of participants experiencing moderate-high self-compassion that decreased then stabilized, 32.0% experiencing moderate self-compassion that decreased then stabilized, 17.3% experiencing high and stable self-compassion across time, and 3.0% experiencing low and decreasing self-compassion. Comparisons of well-being outcomes 1 year post-pandemic indicated that higher levels of self-compassion over time may protect against the impact of initial risk on well-being outcomes. Further work is still needed on heterogeneity in experiences of risk and protective factors during stressful life events.

## Introduction

The recent coronavirus (COVID-19) has quickly spread across the globe since its initial emergence in December 2019. By August 2021, 203 million confirmed cases of COVID-19 were reported globally, including 4.3 million deaths (Shahsavarinia et al., 2022). Although up to 60% of those admitted to hospitals for COVID-19 symptoms report health improvements after discharge (Bagi et al., 2021), the pandemic has also led to unprecedented economic, societal, and political challenges, impacting billions of people globally. As COVID-19 cases have risen, psychological distress related to COVID-19 has also increased due to fear of infection, social isolation, and overburdening of health care institutions (Mann et al., 2020; Mertens et al., 2020). In particular, the negative impacts of the pandemic on well-being, such as psychological distress, have been highlighted in several studies over the last 2 years (e.g., Salari et al., 2020). However, key risk and protective factors that hinder or enhance coping with the challenges and repercussions of the pandemic are less known. Little is also known about individual heterogeneity in such risk and protective factors that may combine to result in better psychological outcomes. Thus, the present paper used 11 waves of data collected over the course of 12 months of the pandemic to identify profiles of risk factors and longitudinal trajectories of self-compassion that may predict well-being outcomes.

### Well-being during the pandemic

Well-being can be defined as flourishing in terms of feelings and in functioning, including elements such as emotional stability and mental health, meaning and satisfaction in life, and self-competence (Huppert and So, 2013). The current pandemic has negatively affected well-being, in particular psychological health. Limcaoco et al. (2020) have found that negative emotions were heightened in the general population during the pandemic across 41 countries. In Hong Kong participants (Tso and Park, 2020), over 65% of survey respondents reported clinically elevated levels of depression, anxiety, and stress. Similarly, in Poland, over 65% of university students indicated mild to severe Generalized Anxiety Disorder symptoms (Rogowska et al., 2020). Particularly due to governmental mandates to reduce the spread of COVID-19 (e.g., lockdowns, social distancing), decreased mental health has been found across demographic groups worldwide (see meta-analysis by Sibley et al., 2020). In the same vein, the pandemic has also impacted another area of well-being: life satisfaction. Increased fear of COVID-19, for example, has been linked to less life satisfaction in several studies (e.g., Satici et al., 2020; Dymecka et al., 2021). Additionally, individuals in lockdown and quarantine have been found to perceive greater social isolation, and in turn, report poorer life satisfaction (Clair et al., 2021; Clark and Lepinteur, 2021).

A less explored element of well-being in relation to the pandemic is perceived control. Perceived control describes the perception that one has the capacity to significantly influence events or outcomes in one’s life (Burger, 1989), thus fitting into the self-competence area of well-being (Huppert and So, 2013). Perceiving high levels of control is considered beneficial as it allows for confidence to positively cope in response to obstacles, including externally caused obstacles such as the pandemic (Seligman and Maier, 1967; Zheng et al., 2020). Indeed, research shows that perceptions of having greater control over oneself and one’s surroundings is related to less anxiety and better life satisfaction during the pandemic (Bidzan et al., 2020; Zheng et al., 2020). Overall, given their relevance to quality of life during the pandemic, in the present work, we focused on three well-being factors: mental health, life satisfaction, and perceived control.

### Risk factors

A number of risk factors have been identified in the literature as being associated with decreased psychological well-being during the COVID-19 pandemic. Broadly, some of the most researched risk factors include (a) sociodemographic factors, (b) cognitive or personality characteristics, and (c) health-related factors (Xiong et al., 2020; Browning et al., 2021). With regards to sociodemographic factors, less education, job loss during the pandemic, lower socioeconomic status, socioeconomic disadvantage, and being a parent have been identified as risk factors for decreased well-being (Lachman and Weaver, 1998; Russell et al., 2020; Zhang et al., 2020; Clair et al., 2021; Fancourt et al., 2021; O’Connor et al., 2021). Cognitive or personality characteristics associated with greater risk for lower well-being during the pandemic include low self-concept clarity, low group identity clarity, less trust in government, more worries about COVID-19, maladaptive personality traits (e.g., less openness and extraversion, more neuroticism), and feeling more lonely or isolated (Lee-Flynn et al., 2011; Greenaway et al., 2015; Fernández et al., 2020; González-Sanguino et al., 2020; Vaswani et al., 2020; Alessandri et al., 2021; Clair et al., 2021; Lee et al., 2021; López-Núñez et al., 2021; Shokrkon and Nicoladis, 2021). With regards to health-related factors, research has identified that less exercise and poorer sleep quality are related to poorer mental health and decreased well-being during and beyond the pandemic (Hertenstein et al., 2019; Franceschini et al., 2020; Rogowska et al., 2020; Zhang et al., 2020; Ahammed et al., 2021; De Sousa et al., 2021). In the present study, we focused on these categories of identified risk factors in relation to the three above-identified elements of well-being (mental health, life satisfaction, perceived control). We additionally took a person-centered approach (as seen in Tisseyre et al., 2021; described further below) to better understand how certain risk factors may co-occur in some individuals.

### Self-compassion

In addition to risk factors, one key protective factor for maintaining high levels of well-being may be self-compassion. Self-compassion has been defined as the ability to accept oneself or one’s suffering with a kind, warm, and non-judgmental attitude (Neff, 2003a), and can also be considered as compassionate self-responding. It is thought that adopting a self-compassionate attitude toward one’s struggles may protect one’s psychological resources in a manner that allows for resilience (Beaumont et al., 2016; Coyne et al., 2020). Studies conducted during the pandemic point to the protective effects of high levels of self-compassion on well-being outcomes (see Waters et al., 2021). Higher self-compassion has been shown to increase tolerance of uncertainty and fear related to the pandemic (Deniz, 2021) and buffer mental health symptoms related to the perceived threat of COVID-19 (Lau et al., 2020) or its associated stressors such as its economic impact (Keng and Hwang, 2022). In a randomized controlled trial, Schnepper et al. (2020) found that 2 weeks of mobile-delivered self-compassion training during a pandemic lockdown reduced more stress in the test group compared to the control group that did not receive such training. It thus seems that people who maintain a highly self-compassionate stance during the pandemic may be better equipped to cope with related challenges, ultimately experiencing more well-being. To test this proposition, we focused on self-compassion trajectories over time during the pandemic and their relation to well-being. We thus used two different types of person-centered analyses in this study, which we describe in the next section.

### Person-centered approach

We examined the roles of sociodemographic, cognitive/personality, and health-related risk factors as well as self-compassion across time using a person-centered framework. Although identifying specific single factors or additive multiple factors related to well-being outcomes during COVID is informative, another useful approach to understanding the relevance of these factors is through person-centered analyses. Person-centered analyses allow for identification of different unobserved subgroups of individuals in the broader sample based on observed factors of interest, thus unveiling heterogeneity with regards to a specific phenomenon (Nylund-Gibson and Choi, 2018). Here, we used two types of person-centered analyses: latent class analysis (LCA) to identify subgroups of the sample that share initial risk factors (as in Lanza and Rhoades, 2013; Tisseyre et al., 2021), and latent class growth analysis (LCGA) to identify subgroups of the sample that share similar patterns of change in self-compassion across the pandemic (Nagin, 1999; Berlin et al., 2014). We were inspired by Nylund-Gibson et al. (2014), who used a latent transition mixture model, which uses a three-step procedure to parsimoniously test the person-level tendency to *move* from one class (in our case, risk class) to another (self-compassion trajectory profile) as well as the interaction effects on potential outcomes of membership in a specific class or profile.

Studies using person-centered approaches in the context of the pandemic have largely focused on heterogeneity in psychological well-being, particularly mental health, in the population. For example, despite the vast number of studies suggesting heightened mental health difficulties during the pandemic, Somé et al. (2022) found that only about one-quarter of their large sample of adults in Canada belonged to a profile of high mental health difficulties. Similarly, Sheeper (2022) found using LCA that over 50% of adults were well adjusted during the pandemic (high life satisfaction, low mental health difficulties), while only 11% were maladjusted on the same metrics. Trajectory analyses of pandemic-related outcomes have also been conducted, with loneliness shown to be heterogeneous in initial levels but stable across the pandemic (Bu et al., 2020), and anxiety and depression decreasing across the pandemic across demographic groups, regardless of initial levels (Fancourt et al., 2021). While these patterns of heterogeneity suggest that only a limited proportion of the population experienced difficulties with well-being during the pandemic, little is yet known about the risk and protective factors that may be associated with these outcomes. Further, existing studies on risk or protective factors have yet to attempt to predict well-being outcomes over time during the pandemic.

### The present study

Thus, using these person-centered approaches, we aimed to distill heterogeneity in the risk factors and self-compassion trajectories that relate to mental health and well-being outcomes approximately 1 year into the pandemic. Given the protective role of self-compassion for well-being, we also assessed whether self-compassion levels across time would buffer the impact of risk factors on outcomes for some subgroups of participants. We aimed to examine (1) how initial risk factors in socioeconomic, cognitive and personality, and health domains at the beginning of the pandemic vary across different subgroups of participants; (2) how longitudinal trajectories in self-compassion throughout the pandemic vary across profiles of participants, and (3) how the varying risk factor subgroups of participants and varying self-compassion trajectories of participants differ on mental health, life satisfaction, and perceived control after 12 months of the pandemic. As part of aim (3), we examined the interaction of risk factor profiles and self-compassion trajectories on the three well-being outcome variables so as to deduce whether self-compassion trajectories would be protective against the negative effects of belonging to a particular risk factor profile at the beginning of the pandemic. Although specific hypotheses regarding model fit or number of classes are not typical in person-centered analyses (Nylund-Gibson and Choi, 2018), based on previous research, we expected that the relatively Low Risk class in the LCA and consistently high self-compassion trajectory profile in the LCGA would experience better well-being outcomes. Furthermore, we expected that those participants in the consistently high self-compassion trajectory profile would be more resilient when facing any combination of risk factors.

## Methods

### Participants

The sample comprised of 3,613 Canadian participants recruited through the polling firm Delvina from a representative web panel of over one-million Canadians. To be included in the study, participants were required to be over 18 years of age and have access to internet on their cell phone, tablet, or computer. Based on Statistics Canada data from 2016, 94% of Canadians have access to internet from home (Statistics Canada, 2016). All participants provided informed consent to participate in the study. Sample sizes varied at each time point, with partial retention of participants at each wave of data collection. In wave 1, the sample was representative of the national population on gender, age, and province of residence: the mean age was 47.65 years (*SD* = 17.01), 50.6% were women, 56.5% were employed, and the average household size was 2.41 persons (*SD* = 1.18). Further detail on the sample can be found in the study technical report by de la Sablonnière et al. (2020).

### Procedures

All study procedures were approved by the ethics committee at the Université de Montréal. Longitudinal data were collected by the polling firm Delvinia over 11 waves, covering 12 months, beginning at the start of the pandemic in April 2020 and ending April 2021. We implemented a rolling cross-sectional survey design (Johnston and Brady, 2002), which allows for dynamic analyses that capture real-time effects of events while lowering participant fatigue and ensuring representativeness of the sample. Our recruitment plan involved initial wave 1 contact with a large pool of respondents: a sample of 250 were drawn every day for 14 days until at least 3,500 (sample size goal) participants were recruited. These same participants were then contacted again for 10 additional waves, following the intervals identified in Supplementary Table 1. Surveys were delivered as an online link to be completed on cell phones, tablets, or computers through the Confirmit platform. Participants were given between 7 to 14 days depending on the wave to complete their survey, in order to ensure maximal re-participation in follow up waves and time for completion. Surveys were approximately 15 to 20 min long at any given wave. Participants were compensated using Delvinia’s point system, redeemable at a store of the participant’s choice. For the present study, participants were compensated with points worth $2.50 CAD per wave. Participants who failed to complete a survey in one wave were still invited to participate in subsequent waves, with missingness handled as described in the next section.

### Planned missingness

In order to improve the validity of data collection, we relied on multi-form designs of planned missingness (for an overview, see Wu and Jia, 2021). Planned missing data designs allow researchers to collect incomplete data from participants by randomly assigning participants to have missing items on a survey. Following best practice procedures, we used several different versions of the questionnaire for which each participant completed two-thirds of the total number of items. This multi-form design is most useful for data collection using a large number of variables balancing time constraints and concerns about respondent burden and fatigue (Rhemtulla et al., 2016). We implemented these designs to collect this large-scale sample and addressed missingness using full information maximum likelihood where possible.

### Measures

#### Risk factors (wave 1, 2, or 3)

Risk factors were measured using a combination of existing measures and new single item measures tailored to the pandemic, as shown in Table 1. When available, risk factors measured at wave 1 were included. If not available at wave 1, we included measures from wave 2 or 3.

**Table 1:** Risk factors and proportion of sample considered at risk.

Risk factor	Sample item	Wave	% at risk	Origin
**Sociodemographic factors**				
Postsecondary education not completed	-	1	19.6	-
Children under 18 in home	How many people in your household are under 18 years old?	1	22.1	Created for study
Job loss during pandemic	Have you lost your job as a result of the COVID-19 crisis?	2	15.7	Created for study
Economically impacted by pandemic	Compared to before the COVID-19 pandemic, my economic situation has improved [or deteriorated].	1	21.5	de la Sablonnière et al. (2009, 2013)
**Cognitive and personality factors**				
Low personal identity clarity	I spend a lot of time wondering about what kind of person I really am.	2	24.1	Campbell et al. (1996)
Low collective identity clarity	I spend a lot of time wondering about what kind of society [province] really is.	2	24.3	Usborne and Taylor (2010)
Low general trust	Generally speaking, would you say that most people can be trusted or that you need to be very careful in dealing with people?	4	33.2	General Social Survey
Maladaptive personality traits	How well do the following statements describe your personality? [Is reserved]	3	24.3	Rammstedt and John (2007)
Worry about virus spread	How concerned are you about the following as they relate to the COVID-19 outbreak? [Getting very sick with the virus]	1	23.0	Montreal Behavioural Medicine Centre
High loneliness	During the past week, because of the COVID-19 crisis, I often felt lonely.	1	29.4	Reynolds et al. (2008)
**Health factors**				
Not exercising indoors	Please indicate the number of times in the last week you engaged in each of the following activities. [Exercising indoors]	1	34.5	Fougeyrollas and Noreau (1998)
Not exercising outdoors	Please indicate the number of times in the last week you engaged in each of the following activities. [Exercising outdoors]	1	33.2	Fougeyrollas and Noreau (1998)
Low sleep quality	How would you describe the quality of your sleep during the last 24 h?	1	20.4	Created for study

Maladaptive personality traits as defined by the DSM-VI includes low extraversion, less agreeableness, and more neuroticism, which are considered maladaptive variants of the Big Five inventory (da Costa et al., 2018).

All binary risk factors were coded as yes (1) or no (0) responses (e.g., having children). All continuous risk factors were initially assessed on a 10-point scale (e.g., maladaptive personality traits). For these continuous risk factors, participant responses were then transformed into binary data points. First, we calculated the frequencies of participant responses, then determined the proportion of the sample that would be considered high risk for that factor at the highest 25% (e.g., 25% of sample reporting lowest ratings of trust). Then, we transformed the participant responses into binary data points by using the highest 25% as a cutoff, assigning higher risk (value of 1) to participants that rated higher than cutoff and assigning lower risk (0) to participants that rated lower than cutoff. For example, if 3 was the cutoff value such that participants rating 1, 2, or 3 were considered high risk while those rating 4 or above were low risk, participant responses 3 or lower were transformed into 1 while those 4 and higher were transformed into 0. The one-quarter cutoff varied as a function of the item; some variable cutoffs consisted of more (e.g., 30%) or less (22%) than one-quarter based on the nearest whole participant response value. Due to the reverse scale for some items, with higher values indicating high risk for some risk factors and low risk for other risk factors, some risk factors listed in Table 1 are positively worded. For all risk factors, these binary codes were then used in the latent class analysis described below.

#### Self-compassion (waves 2 to 10)

We adapted three items from the General Self-Compassion Scale (Neff, 2003b) to assess the positive component of self-compassion during the pandemic at waves 2 through 10 of data collection. At any given wave, participants responded to two of the three items, which included “When something painful happens to me related to the COVID-19 crisis, I try to take a balanced view of the situation,” “when I feel inadequate in my reaction to the current COVID-19 crisis, I try to remind myself that feelings of inadequacy are shared by most people,” “when I do not like my own behavior during the current COVID-19 crisis, I try to be understanding and patient with myself.” Items were rated on a 10-point scale, with 1 indicating strong disagreement and 10 indicating strong agreement. A mean self-compassion score at each wave was calculated by averaging the two items that were answered at that wave. Interitem consistency using McDonald’s ω (*M* = 76.1; Range = 0.68 to 0.80) and Cronbach’s α (*M* = 75.3; Range = 0.68 to 0.79) was satisfactory across waves.

#### Outcomes (wave 11)

##### Mental health

Participants completed a 6-item version of the Short Screening Scales for Non-Specific Psychological Distress (Kessler et al., 2002), which asked if participants felt nervous, hopeless, restless or fidgety, so depressed that nothing could cheer you up, that everything was an effort, and worthless in the past 30 days. Items were rated on a 5-point scale, with 1 indicating having the feeling all of the time, and 5 indicating having the feeling none of the time. Thus, higher scores were indicative of better mental health. Inter-item consistency as indicated by Cronbach’s α was 0.90.

##### Life satisfaction

Participants completed the Satisfaction with Life Scale (SWLS; Diener et al., 1985), a 5-item measure (e.g., “I am satisfied with my life,” “in most ways my life is close to my ideal”). Items were rated on a 10-point scale, with 1 indicating strong disagreement and 10 indicating strong agreement. Inter-item consistency as indicated by Cronbach’s α was 0.92.

##### Perceptions of control

Participants completed the Perceived Personal Control scale (Greenaway et al., 2013), a 3-item measure. Questions included “I feel in control of my life,” “I am free to live my life as I wish” and “my experiences in life are due to my own actions.” Items were rated on a 10-point scale, with 1 indicating strong disagreement and 10 indicating strong agreement. Inter-item consistency as indicated by Cronbach’s α was 0.70.

### Data analysis

The data analytic plan is described below in three sections, outlining (1) the classes of risk factors identified in the sample at wave 1, 2 or 3, (2) the heterogeneous trajectories of self-compassion found in the sample at waves 2 through 10, and (3) the direct and interaction effects of the risk factors and self-compassion trajectories on well-being outcomes at wave 11. All statistical analyses were conducted using Mplus 7.0 and SPSS Statistics 27. Sampling weights were applied to all analyses in order to obtain results on a representative sample of the Canadian population. We relied on a design weight to correct identifiable demographic deviations from population characteristics (Mercer et al., 2018). The weighting process was conducted under the function “calibration” from the *icarus* package in *R*. We identified the best combination of calibration variables and retained the fitting model that minimized the average estimation error on a range of 13 external benchmark measurements based on data available from Statistics Canada. Calibrating with the “logit” method with respect to the variables minor in the household, province of residence, indigenous status and gender led to a reduction of 8.27% of estimation error. The resulting weights range from 0.10 to 3.80 with a mean of 1. A similar weighting process is also reported on in previous work by the author team (Ferrante et al., 2022). Although data was collected at the initial time point from 3,617 participants, four participants did not have sampling weights and were thus dropped from further analysis, resulting in our final sample size of *N* = 3,613.

#### Latent class analysis of risk factors

We conducted latent class analysis (LCA) to identify groups of individuals that may have similar initial risk factors. The 13 risk factors described in Table 1 were used as indicators for class distinction. Between 2 to 6 class models were examined. The best fitting model was determined using the following criteria: (1) smallest Bayesian information criterion (BIC) and sample-size adjusted BIC (aBIC) values, (2) entropy value closest to 1.00, and (3) Vuong-Lo–Mendell–Rubin Likelihood Ratio Test (VLMR LRT) *p*-value showing significantly better likelihood at *k* (number of classes) model compared to the *k*-1 model (Nylund et al., 2007; Berlin et al., 2014). Class size was also considered for determining model fit, but no hard rule was imposed as the sample size was sufficiently large to handle smaller sized classes. Finally, the interpretability and meaningfulness of identified classes in each model was considered in guiding model selection. Once the best fitting model was chosen, classes were named based on relevant indicators present in the respective class. For all classes and profiles (below), results are presented in sample-size descending order.

#### Latent class growth analysis of self-compassion

We examined the profiles of self-compassion across waves 2 to 10 of data collection during the pandemic using Latent Class Growth Analysis (LCGA). For LCGA, the length of time in weeks passed between waves of data varied across the nine waves, which was accounted for in the analyses by setting the times at 0.0, 0.2, 0.4, 0.6, 0.8, 1.2, 1.7, 2.2, and 3.1. Full Information Maximum Likelihood (FIML) estimation was used in Mplus version 8.8, and both linear and quadratic trends were tested, with all within-profile variances for these factors set at 0. Between 2 to 6 profile models were examined, and model fit was assessed using the same criteria as for LCA, by examining BIC, aBIC, entropy, VLMR LRT *p*-values, and profile sizes.

#### Generalized linear model (time 11 outcomes)

As a final step of analyses, we examined the LCGA self-compassion trajectory profiles that were present for each LCA risk factor class, and compared outcomes along the different class by profile groups in an interaction. Although Latent Transition Mixture Modeling (LTMM) using the three-step procedure (as described in Nylund-Gibson et al., 2014) would have been the optimal procedure for this analysis, this model did not converge in the present dataset due to proportions of samples represented in each class by profile group. Thus, we extracted the LCA risk factor classes and LCGA self-compassion profiles into SPSS to (1) examine the proportions of participants that were in each of the class by profile groups, and (2) test the class by profile group interactions in predicting the wave 11 well-being outcomes of perceived control, mental health, and life satisfaction. Due to unequal sample sizes and heterogeneity of variances across groups, we fitted several generalized linear models (GLM) with robust estimation, yielding Wald chi-square statistics for main (class: 4 levels, profile: 4 levels) and interaction effects (4 classes x 4 profiles). GLM automatically drops participants with missing data from analyses. Bonferroni corrections were applied to pairwise comparison *p*-values, accounting for multiple comparisons.

**Table 6:** Sample sizes across cells, risk class by self-compassion trajectory profile.

	Self-compassion trajectories				
	High	Moderately-high	Moderate	Low	Total
**Risk classes**					
Multiple risk	41_a_	157_a_	92_a_	12_a_	302
Cog-Pers and health	125_a_	287_b_	171_ab_	23_ab_	606
SES and Cog-Pers	32_a_	175_b_	144_c_	6_abc_	357
Low risk	329_a_	984_a_	528_a_	38_b_	1879
Total	527	1,603	935	79	3,144
**Wave 11 data available (*N* = 1839)**					
Multiple risk	24	79	47	6^†^	156
Cog-Pers and health	77	173	90	16	356
SES and Cog-Pers	18	111	78	4^†^	211
Low risk	207	586	296	27	1,116
Total	326	949	511	53	1839

^†^This interaction group was eliminated and not considered for further analysis due to its small sample size. Shared subscripts in the same row denote trajectory profile *ns* for which proportions represented in the risk class do not differ significantly from one another at the *p* < 0.05 level.

## Results

### Profiles of risk factors

Model fit statistics for the 2 to 6 class solutions are depicted in Table 2. BIC and entropy mostly continued to increase with each added class, while aBIC was smallest at the 4 class solution. VLMR LRT *p-*values indicated that examining heterogeneity in the sample was warranted, with the 2-class solution showing a significant *p*-value of <0.001. However, the VLMR LRT *p*-values were comparable across the 3- to 6-class solutions with *p*-values > 0.05. Based on aBIC and risk factor distinctions among the classes, we chose the 4-class solution for further examination.

**Table 2:** Model fit statistics for 2 to 6 class models.

Number of classes	BIC	aBIC	Entropy	VLMR LRT (*p*)	Smallest class (%)
2	39037.047	38951.254	0.312	0.002	33.2
3	39016.625	38886.348	0.489	0.574	11.8
4	39032.886	38858.124	0.446	0.788	14.0
5	39074.319	38855.071	0.517	0.780	2.3
6	39139.748	38876.015	0.582	0.760	2.2

Risk factor endorsement proportions by class for the 4-class solution are depicted in Table 3. Classes of risk factors were named based on the elevated risk factors strongly endorsed by participants in that class. Risk factors fell into three domains: SES, cognitive and personality, or health. The SES and Cognitive Risk class (12.2%) showed greater probability of socioeconomic status risk (children in home, economic impact of pandemic) and cognitive and personality risk (low identity clarity, maladaptive personality traits, and high loneliness). The SES and Health Risk class (13.5%) showed elevated probability of socioeconomic risk (low education, economic impact of pandemic) and health risk (low exercise). The Multiple Risk class (25.7%) showed elevated risk across all three risk categories of socioeconomic status (low education, children in home), cognitive and personality (personal identity clarity, high loneliness), and health (sleep problems). The Low Risk class consisted of approximately half of the sample (48.5%) and showed little elevated endorsement across the risk factor categories, although approximately half had children in the home.

**Table 3:** Item endorsement probabilities for each risk indicator by risk class.

	Risk class			
	Multiple risk	Cog-Pers and Health	SES and Cog-pers	Low risk
**Socioeconomic status factors**				
High school education (max)	0.229	0.348	0.151	0.124
Children under 18 in home	**0.533**	0.355	**0.611**	**0.473**
Job loss during pandemic	**0.627**	0.100	0.000	0.098
Economically impacted by pandemic	**0.568**	0.197	0.064	0.159
**Cognitive and personality factors**				
Low personal identity clarity	**0.483**	0.174	**0.977**	0.003
Low collective identity clarity	**0.414**	0.232	**0.486**	0.143
Low general trust	**0.485**	**0.487**	0.219	0.234
Maladaptive personality traits	**0.416**	0.289	**0.450**	0.145
Worry about virus spread	0.379	0.359	0.189	0.144
High loneliness	**0.475**	0.379	0.375	0.179
**Health factors**				
Not exercising indoors	0.229	**0.645**	0.175	0.280
Not exercising outdoors	0.318	**0.621**	0.284	0.214
Low sleep quality	0.376	0.277	0.302	0.139
Overall IEP average	0.426	0.343	0.329	0.180
% of sample	14.3	20.8	14.0	50.9

IEP, Item endorsement probabilities. IEPs over 0.4 are bolded for ease of interpretation. Cog-Pers risk refers to cognitive and personality risk factors domain.

### Longitudinal profiles of self-compassion

Model fit statistics for the 2 to 6 profile solutions are depicted in Table 4. BIC and aBIC continued to decrease in size with each added profile. Entropy peaked at the 4 profile solution, and the VLMR LRT *p*-values were at least marginally significant (<0.10) for the 2 and 4 profile solution. We chose the 4 profile solution for further examination, and for model parsimony, set all nonsignificant parameter means to zero, which modestly improved both BIC (66256.230) and aBIC (66189.504).

**Table 4:** Model fit statistics for 2 to 6 profile models.

Model solution	BIC	aBIC	Entropy	VLMR LRT (*p*)	Smallest profile (%)
2	68171.497	68120.658	0.676	<0.001	46.1
3	66887.997	66824.448	0.705	0.101	15.5
4	66271.127	66194.869	0.715	0.083	3.0
5	66147.253	66058.286	0.666	0.615	1.5
6	66093.761	65992.083	0.672	0.421	1.7

**Figure 1:**
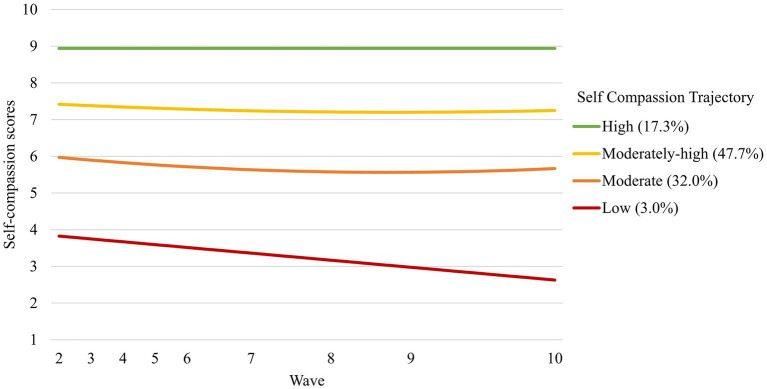
Self-compassion trajectories, 4 profile solution.

The 4-profile solution after adjusting for model parsimony is depicted in Figure 1, and profiles were interpreted with consideration of growth factor estimates, depicted in Table 5. The high self-compassion profile (17.3%) was marked by a high and stable level of self-compassion through waves 2 to 10. The moderately-high self-compassion profile (47.7%) was marked by initially moderately-high self-compassion that decreased significantly and eventually stabilized across time. The moderate self-compassion profile (32.0%) was marked by initially moderate levels of self-compassion that decreased significantly and stabilized across time. Finally, the low self-compassion profile (3.0%) was marked by initially low self-compassion that decreased linearly across time.

**Table 5:** Estimates for self-compassion trajectory profiles.

	High (17.3%)	Moderately-high (47.7%)	Moderate (32.0%)	Low (3.0%)
Intercept	8.94^*^	7.42^*^	5.97^*^	3.83^*^
Slope	0.00	−0.21^*^	−0.40^*^	−0.39^*^
Quadratic	0.00	0.05^*^	0.10^*^	0.00

^*^p < 0.05. Non-significant parameters were set to zero for model parsimony.

### Generalized linear models predicting well-being outcomes

Descriptive statistics for and correlation coefficients among the three wave 11 well-being outcomes are depicted in Supplementary Table 2. We conducted three GLMs with robust estimation and Bonferroni corrections for each of the three well-being outcomes: perceived control, life satisfaction, and mental health. Crosstabs comparisons using chi-squares (χ^2^) showed significant differences in proportions of participants represented across cells, χ^2^(9) = 28.31, *p* < 0.001 (see Table 6). Comparisons of outcome variables are outlined in the next sections in the order of main effects by risk class and self-compassion trajectory profile, then interaction effects of class by profile. Wald χ^2^ statistics for all tests are presented in Table 7.

**Table 7:** Wald χ^2^ tests for all main and interaction effects.

	Main effect: Risk class	Main effect: Self-compassion trajectory	Interaction effect
Perceived control	39.04	118.57	47.54
Life satisfaction	68.81	55.62	21.99
Mental health	70.83	15.98	17.20

All Wald χ^2^ *p*-values < 0.05. Degrees of freedom = 3 for main effects, 9 for interaction effects. Outcome variables were correlated at rs ≤ 0.50, ps < 0.001 (see Supplementary Table 2).

#### Main effects

**Figure 2:**
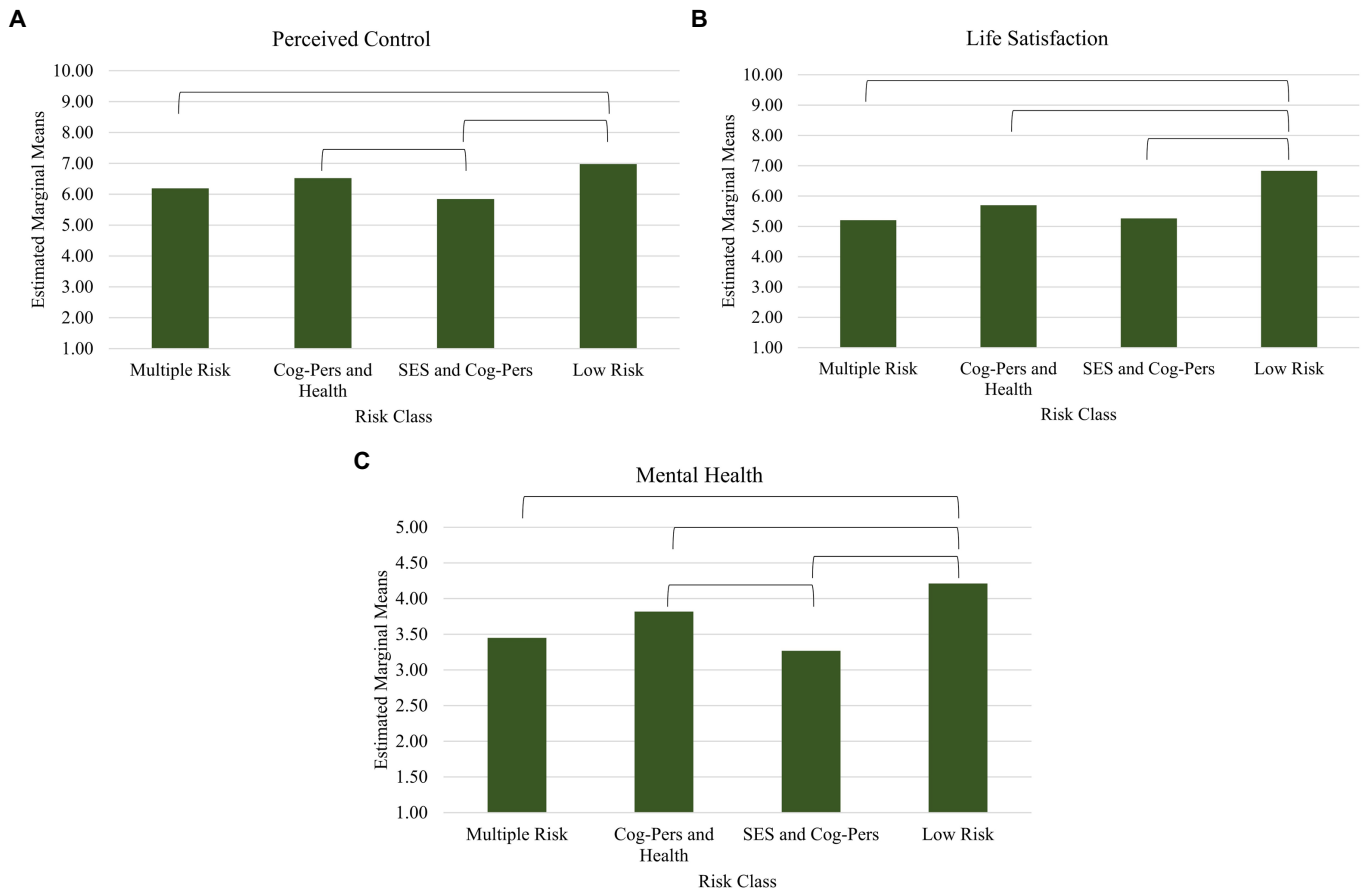
Main effects of risk class on wave 11 outcome variables of Perceived Control **(A)**, Life Satisfaction **(B)**, and Mental Health **(C)**.

Means of all variables by class and trajectory are presented in Table 8. All outcome variables were significantly different in mean levels across the four risk classes, all *p*s < 0.05. *Post-hoc* tests of differences between risk classes are depicted in Figure 2. Participants in the SES and Cognitive-Personality Risk class and Multiple Risk class reported significantly less perceptions of control, lower life satisfaction and more mental health difficulties compared to participants in the Low Risk class. Low Risk class reported higher levels of life satisfaction and less mental health difficulties compared to all other classes. Overall, the Low Risk class tended to show more positive outcomes and less negative outcomes at wave 11 compared to other risk classes.

**Table 8:** Means of outcome variables corresponding to main effects.

	Perceived control		Life satisfaction		Mental health	
	*M*	SD	*M*	SD	*M*	SD
**Risk class**						
Multiple Risk	6.19	0.19	5.20	0.26	3.45	0.16
Cog-Pers and Health	6.52	0.13	5.70	0.20	3.82	0.08
SES and Cog-Pers	5.84	0.13	5.27	0.21	3.27	0.12
Low Risk	6.98	0.14	6.83	0.12	4.21	0.05
**Self-compassion trajectory**						
High	7.43	0.17	6.63	0.21	3.84	0.09
Moderate-high	6.81	0.07	6.19	0.09	3.87	0.04
Moderate	5.89	0.09	5.43	0.11	3.64	0.05
Low	5.40	0.21	4.76	0.32	3.40	0.19

**Figure 3:**
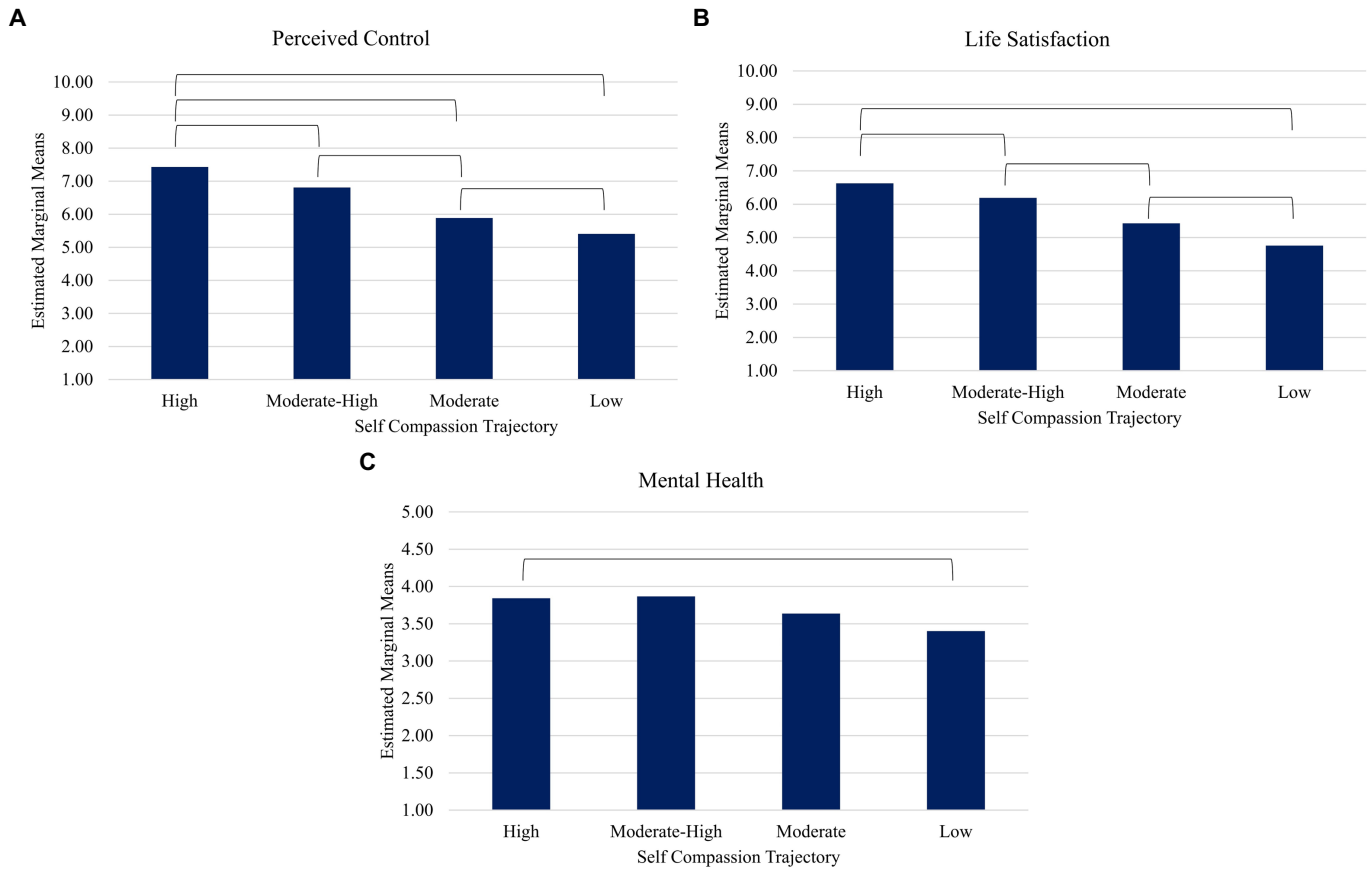
Main effects of self-compassion trajectory profile on wave 11 outcome variables of Perceived Control **(A)**, Life Satisfaction **(B)**, and Mental Health **(C)**.

Similarly, all outcome variables were significantly different in mean levels across the four self-compassion trajectory profiles, all *p*s < 0.05. *Post-hoc* tests of differences between self-compassion trajectory profiles are depicted in Figure 3. Participants who reported high self-compassion across time reported higher perceived control compared to all other profiles. Participants in both the high and moderate-high profiles reported higher life satisfaction compared to those in the moderate and low profiles. For mental health, one significant difference was found: participants in the high self-compassion profile reported fewer mental health difficulties compared to those in the low profile. Overall, the high self-compassion trajectory profile tended to show more positive outcomes at wave 11 compared to other profiles, while the low self-compassion trajectory profile tended to fare worse than other profiles.

##### Interaction effects

**Figure 4:**
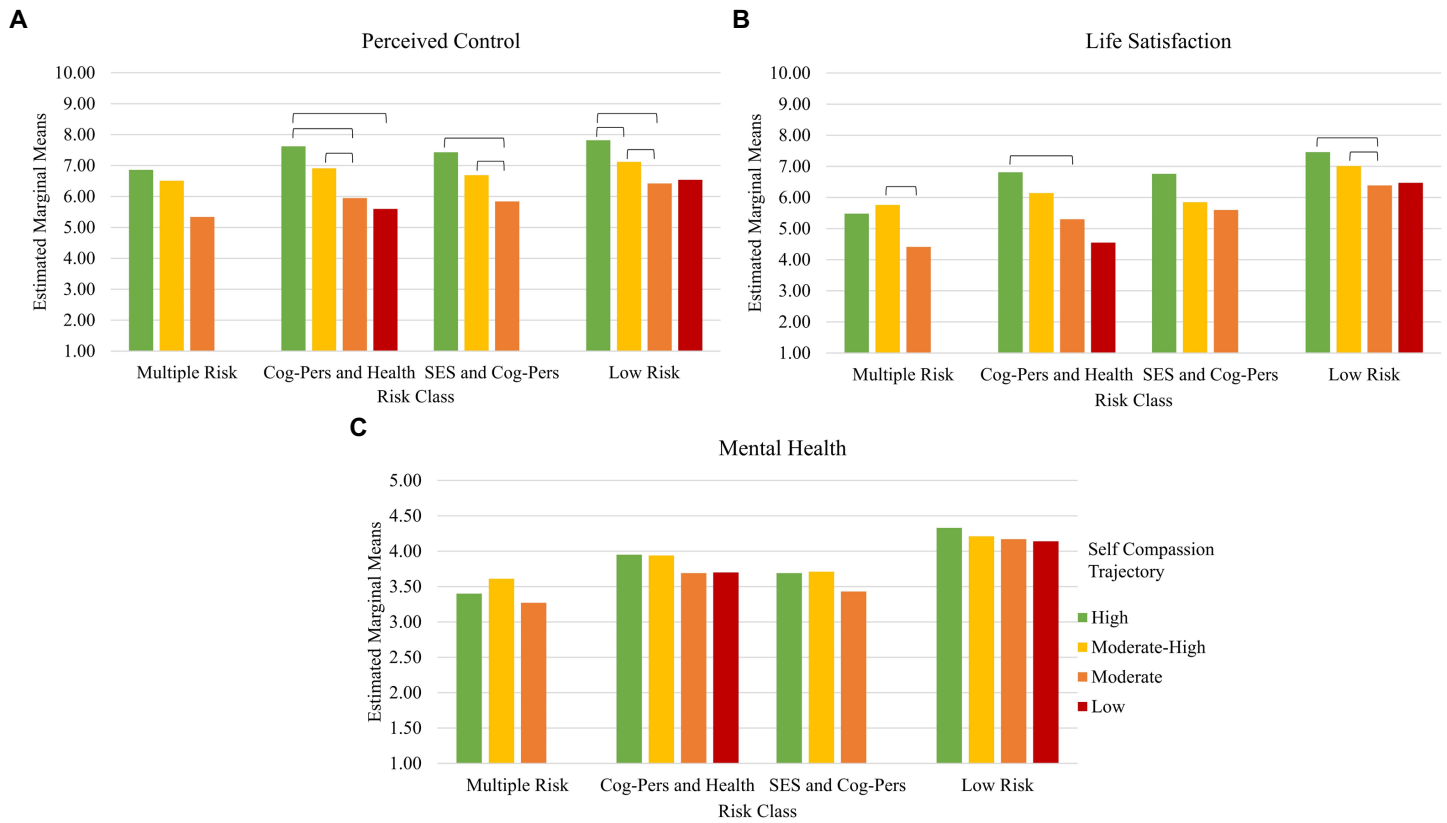
Interaction effects of risk class by self-compassion trajectory profile on wave 11 outcome variables of Perceived Control **(A)**, Life Satisfaction **(B)**, and Mental Health **(C)**.

The overall interaction effects of risk class by self-compassion trajectory profile on all three outcome variables were significant, all *p*s < 0.05. *Post-hoc* tests of differences between trajectories within each risk class are depicted in Figure 4. Participants who belonged to the low self-compassion trajectory profile and Multiple Risk or SES and Cognitive-Personality Risk classes was limited to fewer than 10 participants, and as such the two cells were dropped from *post-hoc* comparisons. All means with the exception of these two cells are depicted in Supplementary Table 3.

In terms of perceived control, having high self-compassion over time served a protective role in most risk classes: in the Cognitive-Personality and Health Risk, SES and Cognitive-Personality Risk, and Low Risk classes, participants with relatively high self-compassion across time often reported higher perceived control compared to those from other self-compassion profiles. Even moderate-high self-compassion across time was protective: in the Cognitive-Personality and Health Risk, SES and Cognitive-Personality Risk, and Low Risk classes, participants with moderate-high self-compassion reported higher perceived control compared to those with only moderate self-compassion. However, no differences arose between risk classes. For life satisfaction, an additive effect in the Low Risk class emerged. Those high or moderate high in self-compassion over time in the Low Risk class reported higher life satisfaction compared to those in the same class reporting moderate levels of self-compassion. Additionally, for life satisfaction in the Cognitive-Personality and Health Risk class, those in the high self-compassion profile reported better life satisfaction compared to those in the moderate self-compassion profile. However, for those in the Multiple Risk class, only having moderate-high self-compassion over time (but not high) was better for life satisfaction compared to the moderate self-compassion profile. No significant *post-hoc* interaction effects of self-compassion by risk class emerged for mental health.

## Discussion

In the present work, we sought to examine heterogeneity in risk factors and self-compassion trajectories that may link to different well-being outcomes later on in the pandemic. With regards to risk factors, we identified four subgroups in our large, nationally representative sample. The Low Risk class consisted of just over 50% of participants, suggesting that most individuals faced relatively low levels of risk in the sociodemographic, cognitive, personality, or health domains that were examined in this study. Meanwhile, 14% of the sample showed heightened risk that cross-cut risk factor domains, 21% showed risks in cognitive or personality and health factors, and 14% showed risks in sociodemographic and cognitive or personality factors. The results point to overall heterogeneity in the risks people faced during the early stages of the pandemic.

Perhaps more importantly, in comparing these risk factor subgroups on selected outcomes in this study, we found some differences in the expected directions. Specifically, the Low Risk class of participants experienced better mental health, more life satisfaction, and higher perceived control compared to most other classes. Similar proportions of the population have been found to experience low mental health, social, and behavioral risk in other studies on COVID-19 (e.g., Curran et al., 2022; Goldstein et al., 2022; Liang et al., 2022). The results point to a potentially important role of key risk factors that occur in the higher risk classes that may set them up for greater impact of stressors such as the pandemic. For example, a closer examination of the IEPs indicates that participants in the Multiple Risk class were more likely to financially suffer from the pandemic (job loss, economic impact). It is possible that those economically impacted by COVID-19 may be particularly at risk for experiencing negative well-being outcomes, echoing previous work highlighting this risk factor during the pandemic (Wilson et al., 2020; Ruengorn et al., 2021). Alternatively, it is possible that specific combinations of elevated risk factors, such as high loneliness and general distrust (in the Cognitive-Personality and Health Risk class) or low identity clarity and maladaptive personality traits (in the SES and Cognitive-Personality Risk class) may create a constellation of risk factors that links to poorer well-being. Indeed, using this perspective, it appears that despite evidence suggesting that parents experienced heightened risk for mental health difficulties during the pandemic (e.g., Russell et al., 2020), our results show that it may be instead those parents who experience concurrent risk factors in the SES, cognitive, or health domains that are particularly at risk for negative well-being outcomes. Other research examining risk factors of pandemic-related mental health outcomes have similarly found that there is high combinatorial variability in risk factors experienced by individuals across populations (Pierce et al., 2021; Curran et al., 2022).

In addition to heterogeneity in risk factors, we found that self-compassion trajectories were variable within the sample, although the great majority of the sample showed moderately-high or moderate self-compassion profiles at the beginning of the pandemic. Echoing existing work on trajectories of worsening mental health and life satisfaction through the pandemic (e.g., Preetz et al., 2021), in these two trajectory profiles, self-compassion decreased across time and then stabilized. On the other hand, approximately 17% of the sample showed high, stable levels of self-compassion across time, indicating that about one-sixth of this nationally representative sample showed resilient kindness toward oneself even during pandemic-related challenges. Meanwhile, only 3% of our sample showed low and linearly decreasing self-compassion across time.

In comparing these self-compassion trajectory profiles on well-being outcomes, we found differences in the expected directions. For perceived control and life satisfaction, participants in the high and stable self-compassion profile showed the most positive outcomes, while those in the low and decreasing self-compassion profile showed the least positive outcomes, paralleling existing work examining self-compassion as a protective factor against mental health difficulties during the pandemic (Chi et al., 2022; Liang et al., 2022). As may be expected, the moderate-high and moderate profiles fell between the high and low profiles, in sequentially decreasing order of positive outcomes. Although mental health outcomes did not differ extensively across profiles, a similar trend as above emerged, with the high, stable profile reporting modestly better mental health relative to other profiles. Similar to past work linking self-compassion with well-being during the pandemic (Lau et al., 2020; Keng and Hwang, 2022), our results point toward a buffering effect of self-compassion on psychological outcomes related to COVID-19.

The finding that one-sixth of our sample (17%) showed high and stable self-compassion is particularly important, as it suggests that some individuals are potentially equipped with the psychological resources to protect themselves from negative outcomes related to the pandemic. Indeed, in our interaction analyses, we found that those who maintained higher levels of self-compassion throughout the pandemic were likely to report higher perceived control across risk factor classes. These findings mirror previous studies demonstrating that higher self-compassion is longitudinally linked to perceptions of greater control over stressful events (Chishima et al., 2018). Further, those who were Low Risk and had higher self-compassion reported more life satisfaction compared to those who were Low Risk but moderate in self-compassion, also echoing past work on the positive association between self-compassion and life satisfaction (Li et al., 2021), potentially due to increased hope that comes with self-compassion (Yang et al., 2016). Thus, even in for those at lower risk of experiencing the negative effects of the pandemic, the protective effects of self-compassion appeared to persist. Overall, these results show similar findings to previous research suggesting that self-compassion as a psychologically protective resource is beneficial even for community members at large who may not experience heightened risk for poor well-being (see meta-analysis on psychological health outcomes by Zessin et al., 2015). Taking a different perspective, it is also possible that those who did not experience heightened economic, personal, and health-related difficulties during the pandemic may have been better prepared to take on the challenges of the pandemic, and thus were able to maintain high levels of self-compassion across time.

Overall, our findings point to heterogeneity in the population in risk factors and self-compassion levels that relate to different well-being outcomes. In particular, they highlight the important protective role of self-compassion during the pandemic, consistent with past work on self-compassion (e.g., Yang et al., 2016; Chishima et al., 2018; Li et al., 2021). Although interaction effects on mental health outcomes did not emerge in our study, we may expect based on previous research that better well-being outcomes should be expected when self-compassion is higher (Lau et al., 2020), even if risk factors are more pronounced. It is possible that our findings of null differences in mental health across risk classes by self-compassion profiles may be due to the generally positive ratings of mental health in our particular sample (average rating of 4.1 on a 5-pt scale).

### Strengths, limitations, and future directions

As a large-scale study of a nationally representative sample of Canadians, this study provides important and valuable information on the well-being of Canadians and the mechanisms or risk factors underlying their outcomes. As such, the sampling and design of the study is a major strength of the paper. Another strength is the use of longitudinal methods. Although some existing works have examined short term changes in well-being and cognitions across the initial few months of the pandemic (e.g., Hiraoka and Tomoda, 2020), our study examined how Canadians fared since the initial months of the pandemic (April 2020) up to and including a full year and 11 timepoints following baseline (April 2021). Additionally, by assessing a number of risk factors covering various domains using latent class analysis, our findings provide information on the relative risk of an array of cognitive, personality, sociodemographic, and health factors that may co-occur during times of heightened stress such as a pandemic. Indeed, a major strength of this paper is the use of data-driven analyses (see Nylund-Gibson and Choi, 2018), which allow for the investigation of individual variability in a given domain, and, particularly relevant to public health, proportions of the population that may be relatively more psychologically affected than others during dramatic events such as the global pandemic.

However, there are several limitations that should be considered in interpreting these findings. First and foremost, there is limited variety in the initial risk factors we examined in this study. In particular, in our deliberations on risk factors to include in the LCA, we excluded age, gender, and ethnoracial background as we did not wish to characterize these biological demographic factors associated with prejudices (-isms) as ‘risk’ factors. However, extensive literature indicates that younger and female individuals report significantly more difficulties related to the pandemic (Bidzan-Bluma et al., 2020; Rogowska et al., 2020), and including these characteristics may have resulted in different risk classes. Further, distinctions in different mental health outcomes were not made in our study, limiting specificity with regards to anxiety or depression symptoms. For example, recent work on mental health effects of the pandemic has shown that job security concerns are related to higher depression while general financial concerns are related to higher anxiety (Wilson et al., 2020). Additionally, although our data were longitudinal, we had only self-reported data and limited number of items (e.g., two items per wave for self-compassion). Furthermore, although we aimed to recruit a nationally representative sample, our sample may still be biased. For example, the mean age of our sample was over 50 years by wave 11, though the mean age in Canada was reported to be 41.7 years in 2021 (Statistics Canada, 2021). Additionally to be untangled are the causal links between risk factors and self-compassion; that is, as earlier alluded, those who experience fewer risk factors may have fewer psychological stressors and thus experience higher and more stable self-compassion. Finally, LCA and LCGA are data-driven approaches, and as such our results are highly dependent on the specific sample and data collected (Nylund-Gibson and Choi, 2018). Thus, the generalizability of our results may be limited, particularly considering the variable retention rates across waves (as low as 50.3%) and factors related to participant attrition that were not assessed in this particular study. However, our longitudinal results of the self-compassion trajectories were reproduced when analyzing only data from participants who had completed three or more waves, lending robustness to our findings.

Building on these limitations, future research may explore different mental health outcomes associated with these risk factors and self-compassion trajectories, including separately examining anxiety and depression symptoms, as well as exploring specific areas of difficulties such as eating disorders or substance use, both of which have increased drastically during the pandemic (Striley and Hoeflich, 2021; Taquet et al., 2022). Additionally, although many of the variables in our study were required to be self-reported (e.g., self-compassion is theoretically only known by the respondent), future work involving observational or clinical assessments of mental health and health-related risk factors such as sleep quality or length may be helpful to increase the validity of these results. Further, although our study was longitudinal, analyses were data-driven. Instead, examining the causal links that may connect different risk factors to well-being over time *via* self-compassion would provide an understanding of the mechanisms that underlie the link between risk factors and well-being. Finally, consideration of other risk factors, trajectories, or outcomes is warranted. For example, risk factors such as experiences with ethnoracial discrimination (Kaushal et al., 2022), stress related to parenting a child during school closures (Hiraoka and Tomoda, 2020; Adams et al., 2021), and loss of social support and social connection increased dramatically during the pandemic (Lee et al., 2020), suggesting these life characteristics may also play important roles in how individuals coped with the pandemic and its impacts.

### Implications and conclusion

In introducing the typologies of social change, de la Sablonnière (2017) proposes that dramatic social change occurs when rapid events lead to profound societal changes, rupturing stability in social structures and altering self-identities. The pandemic is one such dramatic social change that has exerted a forceful and often damaging impact upon the daily lives of many people. Our findings highlight the protective role of self-compassion for alleviating the potentially detrimental mental health and well-being consequences of major life stressors such as the recent pandemic. These findings has clear implications for practice, emphasizing the strengths provided by self-compassion-based programs and interventions to aid individuals affected by life-changing events. In line with our findings and existing work on self-compassion, in recent months, self-compassion-based interventions and preventive programs have gained considerable interest in applied settings (Waters et al., 2021). For example, a novel mobile-based self-compassion program for healthy eating behavior during the pandemic has found that self-compassion was successfully improved in participants after the program and aligned with further improvements in stress and healthy eating (Schnepper et al., 2020). Studies using self-compassion training in combination with other mindfulness-related programs have also provided initial evidence for the beneficial effects of self-compassion in improving well-being outcomes during the pandemic (e.g., González-García et al., 2021). Thus, we may expect that increasing self-compassion may be beneficial for a large proportion of the population in terms of improving well-being outcomes during the pandemic, but also other major life stressors, providing a clear target of intervention for practitioners and clinicians.

Overall, the present work provides important insights on the risk and protective factors that may elevate or buffer the impact of the pandemic on individual emotional and cognitive well-being. In particular, this study points to the need to understand the complex interplay of risk and protective factors that together can inform the extent to which individuals may suffer in the face of chronic, global stressors such as the pandemic. Additionally, the findings highlight that there exist individual differences in the experiences of these factors that must be considered when assessing well-being outcomes. Although the present findings are promising, particularly in light of the links between heightened self-compassion and well-being outcomes, further work is yet needed to better disentangle the multifaceted roles of and individual heterogeneity in risk and protective factors that determine well-being.

## Data availability statement

The raw data supporting the conclusions of this article will be made available by the authors, without undue reservation.

## Ethics statement

The study involved human participants and was reviewed and approved by Comité d’éthique de la recherche en éducation et en psychologie (CEREP) at Université de Montréal. Written informed consent for participation was not required for this study in accordance with the national legislation and the institutional requirements.

## Author contributions

HK, EL, GM, and RdlS conceptualized the study. HK, EL, MP-D, and RdlS contributed to data analysis. HK wrote the initial draft of the manuscript. All authors contributed to the article and approved the submitted version.

## Funding

This research was supported by a grant from the Canadian Institutes of Health Research (Grant #170633). The authors are also grateful for financial support from Centre interdisciplinaire de recherche sur le cerveau et l’apprentissage (CIRCA) and the Centre for the Study of Democratic Citizenship (CSDC).

## Conflict of interest

The authors declare that the research was conducted in the absence of any commercial or financial relationships that could be construed as a potential conflict of interest.

## Publisher’s note

All claims expressed in this article are solely those of the authors and do not necessarily represent those of their affiliated organizations, or those of the publisher, the editors and the reviewers. Any product that may be evaluated in this article, or claim that may be made by its manufacturer, is not guaranteed or endorsed by the publisher.

## References

[ref1] Adams E. L. Smith D. Caccavale L. J. Bean M. K. (2021). Parents are stressed! Patterns of parent stress across COVID-19. Front. Psych. 12:626456. doi: 10.3389/fpsyt.2021.626456, PMID: 33897489 PMC8060456

[ref2] Ahammed B. Jahan N. Seddeque A. Hossain M. T. Khan B. Mamun M. A.. (2021). Exploring the association between mental health and subjective sleep quality during the COVID-19 pandemic among Bangladeshi university students. Heliyon 7:e07082. doi: 10.1016/j.heliyon.2021.e07082, PMID: 34095577 PMC8165399

[ref3] Alessandri G. De Longis E. Golfieri F. Crocetti E. (2021). Can self-concept clarity protect against a pandemic? A daily study on self-concept clarity and negative affect during the COVID-19 outbreak. Identity 21, 6–19. doi: 10.1080/15283488.2020.1846538

[ref4] Bagi H. M. Soleimanpour M. Abdollahi F. Soleimanpour H. (2021). Evaluation of clinical outcomes of patients with mild symptoms of coronavirus disease 2019 (COVID-19) discharged from the emergency department. PLoS One 16:e0258697. doi: 10.1371/journal.pone.0258697, PMID: 34673806 PMC8530279

[ref5] Beaumont E. Durkin M. Martin C. J. H. Carson J. (2016). Compassion for others, self-compassion, quality of life and mental well-being measures and their association with compassion fatigue and burnout in student midwives: a quantitative survey. Midwifery 34, 239–244. doi: 10.1016/j.midw.2015.11.002, PMID: 26628352

[ref6] Berlin K. S. Parra G. R. Williams N. A. (2014). An introduction to latent variable mixture modeling (part 2): longitudinal latent class growth analysis and growth mixture models. J. Pediatr. Psychol. 39, 188–203. doi: 10.1093/jpepsy/jst085, PMID: 24277770

[ref7] Bidzan M. Bidzan-Bluma I. Szulman-Wardal A. Stueck M. Bidzan M. (2020). Does self-efficacy and emotional control protect hospital staff from COVID-19 anxiety and PTSD symptoms? Psychological functioning of hospital staff after the announcement of COVID-19 coronavirus pandemic. Front. Psychol. 11:552583. doi: 10.3389/fpsyg.2020.552583 33424673 PMC7785971

[ref8] Bidzan-Bluma I. Bidzan M. Jurek P. Bidzan L. Knietzsch J. Stueck M.. (2020). A polish and German population study of quality of life, well-being, and life satisfaction in older adults during the COVID-19 pandemic. Front. Psych. 11:585813. doi: 10.3389/fpsyt.2020.585813 PMC7705096 33281646

[ref9] Browning M. H. E. M. Larson L. R. Sharaievska I. Rigolon A. McAnirlin O. Mullenbach L.. (2021). Psychological impacts from COVID-19 among university students: risk factors across seven states in the United States. PLoS One 16:e0245327. doi: 10.1371/journal.pone.0245327, PMID: 33411812 PMC7790395

[ref10] Bu F. Steptoe A. Fancourt D. (2020). Loneliness during a strict lockdown: trajectories and predictors during the COVID-19 pandemic in 38,217 United Kingdom adults. Soc. Sci. Med. 265:113521. doi: 10.1016/j.socscimed.2020.113521, PMID: 33257177 PMC7768183

[ref11] Burger J. M. (1989). Negative reactions to increases in perceived personal control. J. Pers. Soc. Psychol. 56, 246–256. doi: 10.1037/0022-3514.56.2.246

[ref010] Campbell J. D. Trapnell P. D. Heine S. J. Katz I. M. Lavallee L. F. Lehman D. R. (1996). “Self-concept clarity: Measurement, personality correlates, and cultural boundaries”: Correction. Journal of Personality and Social Psychology 70, 141–156. doi: 10.1037/0022-3514.70.6.1114, PMID: 33492565

[ref12] Chi X. Huang L. Zhang J. Wang E. Ren Y. (2022). Latent profiles of multi-dimensionality of self-compassion predict youth psychological adjustment outcomes during the COVID-19: a longitudinal mixture regression analysis. Curr. Psychol., 1–12. doi: 10.1007/s12144-022-03378-3 PMC9273687 35846239

[ref13] Chishima Y. Mizuno M. Sugawara D. Miyagawa Y. (2018). The influence of self-compassion on cognitive appraisals and coping with stressful events. Mindfulness 9, 1907–1915. doi: 10.1007/s12671-018-0933-0

[ref14] Clair R. Gordon M. Kroon M. Reilly C. (2021). The effects of social isolation on well-being and life satisfaction during pandemic. Human. Soc. Sci. Commun. 8, 1–6. doi: 10.1057/s41599-021-00710-3

[ref15] Clark A. E. Lepinteur A. (2021). Pandemic policy and life satisfaction in Europe. Rev. Income Wealth 68, 393–408. doi: 10.1111/roiw.12554 34908597 PMC8661917

[ref16] Coyne L. W. Gould E. R. Grimaldi M. Wilson K. G. Baffuto G. Biglan A. (2020). First things first: parent psychological flexibility and self-compassion during COVID-19. Behav. Anal. Pract. 14, 1092–1098. doi: 10.1007/s40617-020-00435-w 32377315 PMC7200171

[ref17] Curran E. Rosato M. Ferry F. Leavey G. (2022). Prevalence and risk factors of psychiatric symptoms among older people in England during the COVID-19 pandemic: a latent class analysis. Int. J. Ment. Heal. Addict., 1–13. doi: 10.1007/s11469-022-00820-2 PMC9041280 35497074

[ref18] da Costa H. P. Vrabel J. K. Zeigler-Hill V. Vonk J. (2018). DSM-5 pathological personality traits are associated with the ability to understand the emotional states of others. J. Res. Pers. 75, 1–11. doi: 10.1016/j.jrp.2018.05.001

[ref19] de la Sablonnière R. (2017). Toward a psychology of social change: a typology of social change. Front. Psychol. 8:397. doi: 10.3389/fpsyg.2017.00397 28400739 PMC5368273

[ref01] de la Sablonnière R. Auger E. Taylor D. M. Crush J. McDonald D. (2013). Social change in South Africa: A historical approach to relative deprivation. British Journal of Social Psychology, 52, 703–725. doi: 10.1111/bjso.12003 23013238

[ref02] de la Sablonnière R. Taylor D. M. Perozzo C. Sadykova N. (2009). Reconceptualizing relative deprivation in the context of dramatic social change: The challenge confronting the people of Kyrgyzstan. European Journal of Social Psychology 39, 325–345. doi: 10.1002/ejsp.519

[ref20] de la Sablonnière R. Dorfman A. Pelletier-Dumas M. Lacourse É. Lina J. M. Stolle D.. (2020). COVID-19 Canada: The end of the world as we know it? (technical report no. 1). Presenting *The COVID-19 Survey*. Université de Montréal.

[ref21] De Sousa R. A. L. Improta-Caria A. C. Aras-Junior R. de Oliveira E. M. Soci U. P. R. Cassilhas R. C. (2021). Physical exercise effects on the brain during COVID-19 pandemic: links between mental and cardiovascular health. Neurol. Sci. 42, 1325–1334. doi: 10.1007/s10072-021-05082-9, PMID: 33492565 PMC7829117

[ref22] Deniz M. E. (2021). Self-compassion, intolerance of uncertainty, fear of COVID-19, and well-being: a serial mediation investigation. Personal. Individ. Differ. 177:110824. doi: 10.1016/j.paid.2021.110824, PMID: 33723469 PMC7945866

[ref23] Diener E. D. Emmons R. A. Larsen R. J. Griffin S. (1985). The satisfaction with life scale. J. Pers. Assess. 49, 71–75. doi: 10.1207/s15327752jpa4901_13 16367493

[ref24] Dymecka J. Gerymski R. Machnik-Czerwik A. (2021). Fear of COVID-19 as a buffer in the relationship between perceived stress and life satisfaction in the polish population at the beginning of the global pandemic. Health Psychol. Rep. 9, 149–159. doi: 10.5114/hpr.2020.102136 PMC10501413 38084284

[ref25] Fancourt D. Steptoe A. Bu F. (2021). Trajectories of anxiety and depressive symptoms during enforced isolation due to COVID-19 in England: a longitudinal observational study. Lancet Psychiatry 8, 141–149. doi: 10.1016/S2215-0366(20)30482-X, PMID: 33308420 PMC7820109

[ref26] Fernández R. S. Crivelli L. Guimet N. M. Allegri R. F. Pedreira M. E. (2020). Psychological distress associated with COVID-19 quarantine: latent profile analysis, outcome prediction and mediation analysis. J. Affect. Disord. 277, 75–84. doi: 10.1016/j.jad.2020.07.133, PMID: 32799107 PMC7413121

[ref27] Ferrante V. M. Lacourse E. Dorfman A. Pelletier-Dumas M. Lina J.-M. Stolle D.. (2022). COVID-19, economic threat, and identity status: stability and change in prejudice against Chinese people within the Canadian population. Front. Psychol. 13:901352. doi: 10.3389/fpsyg.2022.901352, PMID: 36389476 PMC9650986

[ref020] Fougeyrollas P. Noreau L. Bergeron H. Cloutier R. Dion S. A. St-Michel G. (1998). Social consequences of long term impairments and disabilities: Conceptual approach and assessment of handicap. International Journal of Rehabilitation Research 21, 127–142., PMID: 9924676 10.1097/00004356-199806000-00002

[ref28] Franceschini C. Musetti A. Zenesini C. Palagini L. Scarpelli S. Quattropani M. C.. (2020). Poor sleep quality and its consequences on mental health during the COVID-19 lockdown in Italy. Front. Psychol. 11:574475. doi: 10.3389/fpsyg.2020.574475 33304294 PMC7693628

[ref29] Goldstein E. Brown R. L. Lennon R. P. Zgierska A. E. (2022). Latent class analysis of health, social, and behavioral profiles associated with psychological distress among pregnant and postpartum women during the COVID-19 pandemic in the United States. Birth, 1–11. doi: 10.1111/birt.12664 PMC9349739 35802785

[ref30] González-García M. Álvarez J. C. Pérez E. Z. Fernandez-Carriba S. López J. G. (2021). Feasibility of a brief online mindfulness and compassion-based intervention to promote mental health among university students during the COVID-19 pandemic. Mindfulness 12, 1685–1695. doi: 10.1007/s12671-021-01632-6, PMID: 34025814 PMC8127469

[ref31] González-Sanguino C. Ausín B. Castellanos M. Á. Saiz J. López-Gómez A. Ugidos C.. (2020). Mental health consequences during the initial stage of the 2020 coronavirus pandemic (COVID-19) in Spain. Brain Behav. Immun. 87, 172–176. doi: 10.1016/j.bbi.2020.05.040, PMID: 32405150 PMC7219372

[ref32] Greenaway K. H. Haslam S. A. Cruwys T. Branscombe N. R. Ysseldyk R. Heldreth C. (2015). From “we” to “me”: group identification enhances perceived personal control with consequences for health and well-being. J. Pers. Soc. Psychol. 109, 53–74. doi: 10.1037/pspi0000019, PMID: 25938701

[ref33] Greenaway K. H. Louis W. R. Hornsey M. J. (2013). Loss of control increases belief in precognition and belief in precognition increases control. PLoS One 8:e71327. doi: 10.1371/journal.pone.0071327, PMID: 23951136 PMC3737190

[ref34] Hertenstein E. Feige B. Gmeiner T. Kienzler C. Spiegelhalder K. Johann A.. (2019). Insomnia as a predictor of mental disorders: a systematic review and meta-analysis. Sleep Med. Rev. 43, 96–105. doi: 10.1016/j.smrv.2018.10.006, PMID: 30537570

[ref35] Hiraoka D. Tomoda A. (2020). Relationship between parenting stress and school closures due to the COVID-19 pandemic. Psychiatry Clin. Neurosci. 74, 497–498. doi: 10.1111/pcn.13088, PMID: 32779846 PMC7323183

[ref36] Huppert F. A. So T. T. (2013). Flourishing across Europe: application of a new conceptual framework for defining well-being. Soc. Indic. Res. 110, 837–861. doi: 10.1007/s11205-011-9966-7, PMID: 23329863 PMC3545194

[ref37] Johnston R. Brady H. E. (2002). The rolling cross-section design. Elect. Stud. 21, 283–295. doi: 10.1016/S0261-3794(01)00022-1

[ref38] Kaushal N. Lu Y. Huang X. (2022). Pandemic and prejudice: results from a national survey experiment. PLoS One 17:e0265437. doi: 10.1371/journal.pone.0265437, PMID: 35417461 PMC9007497

[ref39] Keng S.-L. Hwang E. Z. N. (2022). Self-compassion as a moderator of the association between COVID-19 stressors and psychological symptoms: a longitudinal study. Behav. Chang. 39, 263–274. doi: 10.1017/bec.2022.2

[ref40] Kessler R. C. Andrews G. Colpe L. J. Hiripi E. Mroczek D. K. Normand S. L.. (2002). Short screening scales to monitor population prevalences and trends in non-specific psychological distress. Psychol. Med. 32, 959–976. doi: 10.1017/S0033291702006074, PMID: 12214795

[ref41] Lachman M. E. Weaver S. L. (1998). The sense of control as a moderator of social class differences in health and well-being. J. Pers. Soc. Psychol. 74, 763–773. doi: 10.1037/0022-3514.74.3.763, PMID: 9523418

[ref42] Lanza S. T. Rhoades B. L. (2013). Latent class analysis: an alternative perspective on subgroup analysis in prevention and treatment. Prev. Sci. 14, 157–168. doi: 10.1007/s11121-011-0201-1, PMID: 21318625 PMC3173585

[ref43] Lau B. H.-P. Chan C. L.-W. Ng S.-M. (2020). Self-compassion buffers the adverse mental health impacts of COVID-19-related threats: results from a cross-sectional survey at the first peak of Hong Kong’s outbreak. Front. Psych. 11:585270. doi: 10.3389/fpsyt.2020.585270 PMC7674650 33250793

[ref44] Lee C. M. Cadigan J. M. Rhew I. C. (2020). Increases in loneliness among young adults during the COVID-19 pandemic and association with increases in mental health problems. J. Adolesc. Health 67, 714–717. doi: 10.1016/j.jadohealth.2020.08.009, PMID: 33099414 PMC7576375

[ref45] Lee Y. Lui L. M. W. Chen-Li D. Liao Y. Mansur R. B. Brietzke E.. (2021). Government response moderates the mental health impact of COVID-19: a systematic review and meta-analysis of depression outcomes across countries. J. Affect. Disord. 290, 364–377. doi: 10.1016/j.jad.2021.04.050, PMID: 34052584 PMC8159271

[ref46] Lee-Flynn S. C. Pomaki G. DeLongis A. Biesanz J. C. Puterman E. (2011). Daily cognitive appraisals, daily affect, and long-term depressive symptoms: the role of self-esteem and self-concept clarity in the stress process. Pers. Soc. Psychol. Bull. 37, 255–268. doi: 10.1177/0146167210394204, PMID: 21239598

[ref47] Li A. Wang S. Cai M. Sun R. Liu X. (2021). Self-compassion and life-satisfaction among Chinese self-quarantined residents during COVID-19 pandemic: a moderated mediation model of positive coping and gender. Personal. Individ. Differ. 170:110457. doi: 10.1016/j.paid.2020.110457, PMID: 33100455 PMC7576372

[ref48] Liang K. Huang L. Qu D. Bu H. Chi X. (2022). Self-compassion predicted joint trajectories of depression and anxiety symptoms during the COVID-19 pandemic: a five-wave longitudinal study on Chinese college students. J. Affect. Disord. 319, 589–597. doi: 10.1016/j.jad.2022.09.078, PMID: 36155236 PMC9499990

[ref49] Limcaoco R. S. G. Mateos E. M. Fernández J. M. Roncero C. (2020). Anxiety, worry and perceived stress in the world due to the COVID-19 pandemic, march 2020. Preliminary results. medRxiv [Preprint].

[ref50] López-Núñez M. I. Díaz-Morales J. F. Aparicio-García M. E. (2021). Individual differences, personality, social, family and work variables on mental health during COVID-19 outbreak in Spain. Personal. Individ. Differ. 172:110562. doi: 10.1016/j.paid.2020.110562

[ref51] Mann F. D. Krueger R. F. Vohs K. D. (2020). Personal economic anxiety in response to COVID-19. Personal. Individ. Differ. 167:110233. doi: 10.1016/j.paid.2020.110233, PMID: 32834283 PMC7330578

[ref52] Mercer A. Lau A. Kennedy C. (2018). For weighting online opt-in samples, what matters most?. Washington, DC: Pew Research Centre.

[ref53] Mertens G. Gerritsen L. Duijndam S. Salemink E. Engelhard I. (2020). Fear of the coronavirus (COVID-19): predictors in an online study conducted in march 2020. J. Anxiety Disord. 74:102258. doi: 10.1016/j.janxdis.2020.102258, PMID: 32569905 PMC7286280

[ref89] Nagin D. S. (1999). Analyzing developmental trajectories: A semiparametric, group-based approach. Psychological Methods 4, 139–157. doi: 10.1037/1082-989X.4.2.139 11285809

[ref54] Neff K. D. (2003a). Self-compassion: an alternative conceptualization of a healthy attitude toward oneself. Self Identity 2, 85–101. doi: 10.1080/15298860309032

[ref55] Neff K. D. (2003b). The development and validation of a scale to measure self-compassion. Self Identity 2, 223–250. doi: 10.1080/15298860309027

[ref030] Nylund K. L. Asparouhov T. Muthén B. O. (2007). Deciding on the number of classes in latent class analysis and growth mixture modeling: A monte carlo simulation study. Structural Equation Modeling: A Multidisciplinary Journal 14, 535–569. doi: 10.1080/10705510701575396, PMID: 32834283

[ref56] Nylund-Gibson K. Choi A. Y. (2018). Ten frequently asked questions about latent class analysis. Transl. Iss. Psychol. Sci. 4, 440–461. doi: 10.1037/tps0000176

[ref57] Nylund-Gibson K. Grimm R. Quirk M. Furlong M. (2014). A latent transition mixture model using the three-step specification. Struct. Equ. Model. Multidiscip. J. 21, 439–454. doi: 10.1080/10705511.2014.915375

[ref58] O’Connor R. C. Wetherall K. Cleare S. McClelland H. Melson A. J. Niedzwiedz C. L.. (2021). Mental health and well-being during the COVID-19 pandemic: longitudinal analyses of adults in the UK COVID-19 Mental Health & Wellbeing study. Br. J. Psychiatry 218, 326–333. doi: 10.1192/bjp.2020.212, PMID: 33081860 PMC7684009

[ref59] Pierce M. McManus S. Hope H. Hotopf M. Ford T. Hatch S. L.. (2021). Mental health responses to the COVID-19 pandemic: a latent class trajectory analysis using longitudinal UK data. Lancet Psychiatry 8, 610–619. doi: 10.1016/S2215-0366(21)00151-6, PMID: 33965057 PMC9764381

[ref60] Preetz R. Filser A. Brömmelhaus A. Baalmann T. Feldhaus M. (2021). Longitudinal changes in life satisfaction and mental health in emerging adulthood during the COVID-19 pandemic. Risk Protect. Factors Emerg. Adulthood 9, 602–617. doi: 10.1177/21676968211042109

[ref040] Rammstedt B. John O. P. (2007). Measuring personality in one minute or less: A 10 item short version of the Big Five Inventory in English and German. J Res. Pers. 41, 203–212. doi: 10.1016/j.jrp.2006.02.001

[ref070] Reynolds D. Garay J. Deamond S. Moran M. Gold W. Styra R. (2008). Understanding, compliance and psychological impact of the SARS quarantine experience. Epidemiology and Infection 136, 997–1007. doi: 10.1017/S0950268807009156, PMID: 17662167 PMC2870884

[ref61] Rhemtulla M. Savalei V. Little T. D. (2016). On the asymptotic relative efficiency of planned missingness designs. Psychometrika 81, 60–89. doi: 10.1007/s11336-014-9422-0, PMID: 25217133

[ref62] Rogowska A. M. Kunierz C. Bokszczanin A. (2020). Examining anxiety, life satisfaction, general health, stress and coping styles during COVID-19 pandemic in polish sample of university students. Psychol. Res. Behav. Manag. 13, 797–811. doi: 10.2147/PRBM.S266511, PMID: 33061695 PMC7532061

[ref63] Ruengorn C. Awiphan R. Wongpakaran N. Wongpakaran T. Nochaiwong S. (HOME-Survey), H. O. and M. H. C. E. S. R. G (2021). Association of job loss, income loss, and financial burden with adverse mental health outcomes during coronavirus disease 2019 pandemic in Thailand: a nationwide cross sectional study. Depress. Anxiety 38, 648–660. doi: 10.1002/da.23155, PMID: 33793028 PMC8251094

[ref64] Russell B. S. Hutchison M. Tambling R. Tomkunas A. J. Horton A. L. (2020). Initial challenges of caregiving during COVID-19: caregiver burden, mental health, and the parent-child relationship. Child Psychiatry Hum. Dev. 51, 671–682. doi: 10.1007/s10578-020-01037-x, PMID: 32749568 PMC7398861

[ref65] Salari N. Hosseinian-Far A. Jalali R. Vaisi-Raygani A. Rasoulpoor S. Mohammadi M.. (2020). Prevalence of stress, anxiety, depression among the general population during the COVID-19 pandemic: a systematic review and meta-analysis. Glob. Health 16, 1–11. doi: 10.1186/s12992-020-00589-w PMC7338126 32631403

[ref66] Satici B. Gocet-Tekin E. Deniz M. E. Satici S. A. (2020). Adaptation of the fear of COVID-19 scale: its association with psychological distress and life satisfaction in Turkey. Int. J. Ment. Heal. Addict., 1–9. doi: 10.1007/s11469-020-00294-0 PMC7207987 32395095

[ref67] Schnepper R. Reichenberger J. Blechert J. (2020). Being my own companion in times of social isolation - a 14-day mobile self-compassion intervention improves stress levels and eating behavior. Front. Psychol. 11:2645. doi: 10.3389/fpsyg.2020.595806 PMC7581850 33162922

[ref68] Seligman M. E. Maier S. F. (1967). Failure to escape traumatic shock. J. Exp. Psychol. 74, 1–9. doi: 10.1037/h0024514, PMID: 6032570

[ref69] Shahsavarinia K. Amiri P. Mousavi Z. Gilani N. Saadati M. Soleimanpour H. (2022). Prediction of PTSD related to COVID-19 in emergency staff based on the components of self-compassion and perceived social support. BMC Psychiatry 22, 1–10. doi: 10.1186/s12888-022-04017-8 35641937 PMC9154198

[ref70] Sheeper K. N. (2022). Using latent profile analysis and latent class analysis to link Covid-related mitigation behaviors and mental health. San Diego, CA: San Diego State University.

[ref71] Shokrkon A. Nicoladis E. (2021). How personality traits of neuroticism and extroversion predict the effects of the COVID-19 on the mental health of Canadians. PLoS One 16:e0251097. doi: 10.1371/journal.pone.0251097, PMID: 34010299 PMC8133466

[ref72] Sibley C. G. Greaves L. M. Satherley N. Wilson M. S. Overall N. C. Lee C. H. J.. (2020). Effects of the COVID-19 pandemic and nationwide lockdown on trust, attitudes toward government, and well-being. Am. Psychol. 75, 618–630. doi: 10.1037/amp0000662, PMID: 32496074

[ref73] Somé N. H. Wells S. Felsky D. Hamilton H. A. Ali S. Elton-Marshall T.. (2022). Self-reported mental health during the COVID-19 pandemic and its association with alcohol and cannabis use: a latent class analysis. BMC Psychiatry 22, 1–13. doi: 10.1186/s12888-022-03917-z 35490222 PMC9055215

[ref74] Statistics Canada. (2016). Census profile, 2016. Catalogue Number: 98-316-X2016001. Available at: https://www150.statcan.gc.ca/n1/en/catalogue/98-316-X2016001

[ref75] Statistics Canada. (2021). Canada’s population estimates: Age and sex, July 2021, 2021. Available at: https://www150.statcan.gc.ca/n1/daily-quotidien/210929/dq210929d-eng.htm

[ref76] Striley C. W. Hoeflich C. C. (2021). Converging public health crises: substance use during the coronavirus disease 2019 pandemic. Curr. Opin. Psychiatry 34, 325–331. doi: 10.1097/YCO.0000000000000722, PMID: 34001699 PMC8183237

[ref77] Taquet M. Geddes J. R. Luciano S. Harrison P. J. (2022). Incidence and outcomes of eating disorders during the COVID-19 pandemic. Br. J. Psychiatry 220, 262–264. doi: 10.1192/bjp.2021.105, PMID: 35048812 PMC7612698

[ref91] Tisseyre L. Lacourse E. Labelle R. Paquin S. Herba C. M. (2021). A person-centered approach to studying associations between psychosocial vulnerability factors and adolescent depressive symptoms and suicidal ideation in a Canadian longitudinal sample. Development and Psychopathology 33, 351–362. doi: 10.1017/s0954579420000012, PMID: 32381149

[ref78] Tso I. F. Park S. (2020). Alarming levels of psychiatric symptoms and the role of loneliness during the COVID-19 epidemic: a case study of Hong Kong. Psychiatry Res. 293:113423. doi: 10.1016/j.psychres.2020.113423, PMID: 32871487 PMC7443338

[ref94] Usborne E. Taylor D. M. (2010). The role of cultural identity clarity for self-concept clarity, self-esteem, and subjective well-being. Personality and Social Psychology Bulletin 36, 883–897. doi: 10.1177/0146167210372215, PMID: 20519575

[ref79] Vaswani M. Alviar L. Giguere B. (2020). Can cultural identity clarity protect the well-being of Latino/a Canadians from the negative impact of race-based rejection sensitivity? Cultur. Divers. Ethnic Minor. Psychol. 26, 347–355. doi: 10.1037/cdp0000302, PMID: 31436442

[ref80] Waters L. Algoe S. B. Dutton J. Emmons R. Fredrickson B. L. Heaphy E.. (2021). Positive psychology in a pandemic: buffering, bolstering, and building mental health. J. Posit. Psychol. 17, 1–21. doi: 10.1080/17439760.2021.1871945

[ref81] Wilson J. M. Lee J. Fitzgerald H. N. Oosterhoff B. Sevi B. Shook N. J. (2020). Job insecurity and financial concern during the COVID-19 pandemic are associated with worse mental health. J. Occup. Environ. Med. 62, 686–691. doi: 10.1097/JOM.0000000000001962, PMID: 32890205

[ref83] Wu W. Jia F. (2021). Applying planned missingness designs to longitudinal panel studies in developmental science: an overview. New Dir. Child Adolesc. Dev. 2021, 35–63. doi: 10.1002/cad.20391, PMID: 33470035

[ref84] Xiong J. Lipsitz O. Nasri F. Lui L. M. W. Gill H. Phan L.. (2020). Impact of COVID-19 pandemic on mental health in the general population: a systematic review. J. Affect. Disord. 277, 55–64. doi: 10.1016/j.jad.2020.08.001, PMID: 32799105 PMC7413844

[ref85] Yang Y. Zhang M. Kou Y. (2016). Self-compassion and life satisfaction: the mediating role of hope. Personal. Individ. Differ. 98, 91–95. doi: 10.1016/j.paid.2016.03.086

[ref86] Zessin U. Dickhäuser O. Garbade S. (2015). The relationship between self compassion and well being: a meta analysis. Appl. Psychol. 7, 340–364. doi: 10.1111/aphw.12051, PMID: 26311196

[ref87] Zhang S. X. Wang Y. Rauch A. Wei F. (2020). Unprecedented disruption of lives and work: health, distress and life satisfaction of working adults in China one month into the COVID-19 outbreak. Psychiatry Res. 288:112958. doi: 10.1016/j.psychres.2020.112958, PMID: 32283450 PMC7146665

[ref88] Zheng L. Miao M. Gan Y. (2020). Perceived control buffers the effects of the COVID 19 pandemic on general health and life satisfaction: the mediating role of psychological distance. Appl. Psychol. 12, 1095–1114. doi: 10.1111/aphw.12232, PMID: 32955170 PMC7537495

