# Supplementary material for: Initial risk factors, self-compassion trajectories, and well-being outcomes during the COVID-19 pandemic: A person-centered approach
Source: Front Psychol. 2023 Feb 8;13:1016397. doi: 10.3389/fpsyg.2022.1016397 (PMC9945549; doi:10.3389/fpsyg.2022.1016397)
Supplement: Supplementary file 1 [file Data_Sheet_1.pdf]

Initial risk factors, self-compassion trajectories, and well-being outcomes during the COVID-19 pandemic: A person-centered approach

Supplementary Tables

Table 1. Timetable for 11 waves of data collection

Wave	Starting date	Intervals	*N*	% Respondents	% Women	Mean age
1	April 6, 2020	-	3617	100%	50.5%	47.65
2	April 21, 2020	2 weeks	2282	63.0%	48.9%	49.03
3	May 4, 2020	2 weeks	2369	65.5%	49.2%	48.81
4	May 18, 2020	2 weeks	2296	63.5%	48.5%	48.90
5	June 1, 2020	2 weeks	2154	59.6%	48.7%	49.32
6	June 15, 2020	2 weeks	2116	58.5%	48.8%	49.36
7	July 13, 2020	4 weeks	2072	57.6%	49.1%	49.80
8	August 17, 2020	5 weeks	1871	51.7%	49.4%	50.42
9	Sept. 21, 2020	6 weeks	1821	50.3%	48.4%	51.82
10	Nov. 26, 2020	10 weeks	1883	52.5%	48.4%	50.30
11	April 13, 2021	20 weeks	2002	55.4%	49.5%	51.15

Note. Percentages of respondents in each wave are calculated based on the wave 1 sample. Intervals indicate the number of weeks since previous wave of data collection. Four participants did not have sampling weights and were thus dropped from the present manuscript, resulting in final *N* = 3613 in the present study.

Table 2. Descriptive statistics and correlations for the Wave 11 well-being variables

	2	3	Mean	SD	IQR 25%	IQR 75%
1. Perceived Control	.50	.37	6.84	1.76	5.67	8.00
2. Life Satisfaction		.47	6.46	2.07	5.20	8.00
3. Mental Health			4.01	.89	3.50	4.67

Note. All correlations significant at $p < .001$.

Table 3. Means of outcome variables corresponding to interaction effects (Figures 2-4)

	Risk Class							
	Multiple Risk		Cog-Pers and Health		SES and Cog-Pers		Low Risk	
	M	SD	*M*	SD	*M*	SD	*M*	SD
Self Compassion Trajectory				Perceived Control				
High	6.86	.50	7.62	.20	7.43	.38	7.82	.12
Moderate-High	6.51	.21	6.91	.13	6.69	.13	7.12	.06
Moderate	5.34	.26	5.95	.18	5.84	.18	6.42	.09
Low			5.60	.41			6.54	.53
				Life Satisfaction				
High	5.48	.59	6.81	.26	6.76	.50	7.46	.14
Moderate-High	5.76	.24	6.14	.17	5.85	.20	7.01	.07
Moderate	4.41	.28	5.30	.24	5.60	.18	6.39	.10
Low			4.55	.68			6.47	.44
				Mental Health				
High	3.40	.22	3.95	.10	3.69	.25	4.33	.05
Moderate-High	3.61	.11	3.94	.07	3.71	.09	4.21	.03
Moderate	3.27	.12	3.69	.12	3.43	.13	4.17	.05
Low			3.70	.28			4.14	.20
